# What is the most appropriate knowledge synthesis method to conduct a review? Protocol for a scoping review

**DOI:** 10.1186/1471-2288-12-114

**Published:** 2012-08-03

**Authors:** Monika Kastner, Andrea C Tricco, Charlene Soobiah, Erin Lillie, Laure Perrier, Tanya Horsley, Vivian Welch, Elise Cogo, Jesmin Antony, Sharon E Straus

**Affiliations:** 1 Li Ka Shing Knowledge Institute of St. Michael’s hospital, 209 Victoria Street, Toronto, ON, M5B 1W8, Canada; 2 Continuing Education and Professional Development, Faculty of Medicine, University of Toronto, Toronto, ON, Canada; 3 Royal College of Physicians and Surgeons of Canada, Ottawa, ON, Canada; 4 Centre for Global Health, Institute of Population Health, University of Ottawa, Ottawa, ON, Canada; 5 Department of Medicine, Faculty of medicine, University of Toronto, Toronto, ON, Canada

## Abstract

**Background:**

A knowledge synthesis attempts to summarize all pertinent studies on a specific question, can improve the understanding of inconsistencies in diverse evidence, and can identify gaps in research evidence to define future research agendas. Knowledge synthesis activities in healthcare have largely focused on systematic reviews of interventions. However, a wider range of synthesis methods has emerged in the last decade addressing different types of questions (e.g., realist synthesis to explore mediating mechanisms and moderators of interventions). Many different knowledge synthesis methods exist in the literature across multiple disciplines, but locating these, particularly for qualitative research, present challenges. There is a need for a comprehensive manual for synthesis methods (quantitative/qualitative or mixed), outlining how these methods are related, and how to match the most appropriate knowledge synthesis method to answer a research question. The objectives of this scoping review are to: 1) conduct a systematic search of the literature for knowledge synthesis methods across multi-disciplinary fields; 2) compare and contrast the different knowledge synthesis methods; and, 3) map out the specific steps to conducting the knowledge syntheses to inform the development of a knowledge synthesis methods manual/tool.

**Methods:**

We will search relevant electronic databases (e.g., MEDLINE, CINAHL), grey literature, and discipline-based listservs. The scoping review will consider all study designs including qualitative and quantitative methodologies (excluding economic analysis or clinical practice guideline development), and identify knowledge synthesis methods across the disciplines of health, education, sociology, and philosophy. Two reviewers will pilot-test the screening criteria and data abstraction forms, and will independently screen the literature and abstract the data. A three-step synthesis process will be used to map the literature to our objectives.

**Discussion:**

This project represents the first attempt to broadly and systematically identify, define and classify knowledge synthesis methods (i.e., less traditional knowledge synthesis methods). We anticipate that our results will lead to an accepted taxonomy for less traditional knowledge synthesis methods, and to the development and implementation of a methods manual for these reviews which will be relevant to a wide range of knowledge users, including researchers, funders, and journal editors.

## Background

Knowledge synthesis has the potential to inform the management of health problems [1] and is integral to the health of the Canadian population [2]. A knowledge synthesis summarizes all pertinent studies on a specific question, can improve the understanding of inconsistencies in diverse evidence, and can define future research agendas [1,3]. Knowledge synthesis is also an important part of the knowledge translation (KT) process (and ideally should form the ‘base unit’ of KT strategies for providers and policy makers), and be used to provide the evidence base for KT products including clinical practice guidelines, policy briefs and decision aids [4]. As such, knowledge synthesis can be used to interpret results of individual studies within the context of the totality of evidence. This is an important consideration, given that basing practice and policy decisions on a single study or expert opinion can be misleading [5].

Knowledge synthesis activities in healthcare have often focused on the methodologically rigorous Cochrane reviews, most commonly of interventions. The definition of a systematic review according to the Cochrane Collaboration is “A review of clearly formulated questions that uses systematic and explicit methods to identify, select, and critically appraise relevant research, and to collect and analyse data from the studies that are included in the review. Statistical methods (meta-analysis) may or may not be used to analyse and summarise the results of the included studies” [6]. However, Cochrane-like review methods may not always be applicable for answering all knowledge synthesis questions, particularly those investigating complex and multidisciplinary topics [7,8]. For example, members of our team recently attempted to conduct a systematic review to better understand the relationship between the perceived characteristics of clinical practice guidelines and their uptake by clinicians, and found that a flexible approach that borrowed relevant components of less traditional knowledge synthesis methods (i.e., including realist reviews and meta-ethnography) was more relevant to determine the mechanisms and circumstances underpinning guideline implementation [9]. This example highlights the need for less traditional methods for completing a review. By matching the appropriate design to fit the question, synthesis outputs are more likely to be relevant and be useful for end users.

Furthermore, a traditional review such as a Cochrane review cannot always explain why particular interventions work in some settings but not in others [10]. For example, a Cochrane review found that school feeding programs significantly improved the growth and cognitive performance of disadvantaged children [11], but failed to provide direction for policy-makers to decide which intervention should be implemented and under what circumstances. By conducting a realist review alongside the Cochrane review (which can be used to understand ‘what works for whom and under what circumstances’ [10]), the authors were able to provide concrete recommendations that could be implemented in practice and policy making [7]. To address these types of questions and adequately incorporate the needs, preferences and experiences of patients into healthcare delivery, there is an increasing need to consider less traditional review methods of complex evidence (i.e., heterogeneous, methodologically diverse, difficult to classify, and contradictory) [12,13]. Another approach is to consider conducting a systematic review as a “first step” to better understand complex evidence (or to conduct them in parallel with novel reviews), particularly for evidence generated from philosophy and the social sciences. The increasing number of synthesis methods that have recently emerged within the healthcare literature supports this need [14-17].

Table 1 summarizes a selection of knowledge synthesis methods that currently exist in the literature across multiple disciplines (identified through consultation with knowledge synthesis experts and qualitative researchers). Although many of these approaches can be applied to healthcare situations, the methods for conducting them have not been as clearly operationalized as traditional reviews of interventions. Consultation with researchers and end users of reviews that we conducted in preparation for this research indicate a lack of clarity around how to match the appropriate review method to the research question, the methods used to conduct these reviews, and how to analyze and present the results from the review to inform decision making. These issues are challenging for researchers interested in tackling reviews of complex questions and for decision makers trying to interpret and apply this evidence. Other identified challenges involve locating the numerous synthesis methods (particularly those for synthesizing qualitative research), which can be problematic and resource-intensive since they are scattered widely within the literature and across many different disciplines and databases. The terms used to describe the different synthesis methods are often similar (e.g., ‘meta-synthesis’, ‘meta-ethnography’, ‘meta-narrative’, ‘meta-study’, ‘meta-interpretation’) and their definitions can overlap [12]. This area of research is further complicated because some of these methods are referred to as a ‘complete’ synthesis method (i.e., providing guidance on the search strategy, study selection, appraisal, and analysis), while others provide guidance only on specific parts of the process, such as data analysis [12].

**Table 1:** Characteristics of a preliminary list of existing knowledge synthesis methods

**Knowledge synthesis method**	**Type of evidence**	**Description**
**Bayesian meta-analysis** *(Sutton, AJ, 2001; Roberts KA, 2002) *[18,19]	Mixed (qualitative and quantitative)	A method used in meta-analysis to offer flexibility in handling data from diverse study types (i.e., the integration qualitative and quantitative forms of evidence). It allows qualitative evidence to contribute to meta-analysis by identifying variables to be included and providing evidence about effect sizes (qualitative evidence gets converted into quantitative form); and helps to ensure that meta-analyses more properly reflect the diversity of evidence at primary level – it recognizes the fact that evidence from multiple sources usually needs to be combined to inform policy decisions.
**Content analysis** *(Stemler S, 2001) *[20]	Qualitative	A technique for categorising data and determining the frequencies of these categories. It differs from more ‘qualitative’ methods in that it requires categorization to be sufficiently precise to allow multiple coders to achieve the same results, it relies on the systematic application of rules, and it tends to draw on the concepts of validity and reliability. Text is condensed into fewer content-related categories.
**Critical interpretive synthesis** *(Dixon-Woods, 2006) *[21]	Mixed	Developed from meta-ethnography, it is an approach to the entire process of a review rather than just the synthesis component. It uses an iterative approach to refining the research question, the searching and selection of articles from the literature, and defining and applying codes and categories.
**Cross-design synthesis** *(Droitcour J, 1993) *[22]	Mixed	A form of meta-analysis, which allows the mixing of different quantitative research designs (e.g. randomized controlled trials and observational studies) and the pooling of evidence using modeling to estimate a ‘true’ effect of a policy or programme, conditional on both the design of the study and the characteristics of the relevant population
**Ecological triangulation** *(Banning, date unknown) *[23]	Qualitative	Uses the concept of triangulation, in which phenomena are studied from a variety of vantage points. The method ‘unpicks’ the mutually interdependent relationships between behaviour, persons, and environments, and requires ‘ecological sentences’ to be formulated during synthesis: “With this intervention, these outcomes occur with these population foci and within these ages with these genders… and these ethnicities in these settings”.
**Framework synthesis** *(Pope, 2000; Brunton, 2006) *[24,25]	Qualitative	Offers a highly structured approach to organizing and analysing data (i.e., indexing using numerical codes, rearranging data into charts, etc) to handle the large volume of information resulting from qualitative research. It’s distinct from other methods in that it utilises an ‘a priori’ framework informed by background material and team discussions to extract and synthesize findings (i.e., a deductive approach). The ‘synthetic’ product may be expressed in the form of a chart for each key dimension, which can be used to map the nature and range of the concept under study.
**Grounded theory** *(Strauss & Corbin, 1998) *[26]	Qualitative	A primary research approach used as a method for qualitative sampling, data collection and analysis. It offers the ‘constant comparative method’ (the most widely used element of grounded theory) to be used to identify patterns and iterations in primary data. It is an inductive approach to analysis, allowing the theory to emerge from the data.
**Interpretive Synthesis/Integrative synthesis** *(Noblit and Hare (1988) *[27]	Qualitative	Noblit and Hare (1988) distinguish between the approaches of ‘interpretive’ and ‘integrative’ forms of synthesis which can be described as exploring the nature of the synthesis rather than its application. Interpretive synthesis combines evidence with an intent to develop new concepts and theories (interpretations).
**Meta-ethnography** *(Noblit G & Hare R., 1988) *[27]	Qualitative	A novel synthesis method aimed to uncover a new theory to explain the range of research findings encountered. It is a way of re-analysing and comparing the texts of published studies (rather than the original data of each) to produce a new interpretation. The approach involves induction and interpretation in which separate parts are brought together to forma a “whole” (i.e., looking for new theory or ‘line of argument’ to explain all the studies) so that the result is greater than the sum of its parts. The product is the translation of studies into one another, which encourages the researcher to understand and transfer ideas, concepts and metaphors across different studies.
**Meta-interpretation** *(Weed, 2005) *[28]	Qualitative	A method that follows an ideographic rather than pre-determined approach to the development of the following components: exclusion criteria, a focus on meaning in context, interpretations as raw data for synthesis, an iterative approach to the theoretical sampling of studies for synthesis, and a transparent audit trail demonstrating the trustworthiness of the synthesis
**Meta-narrative** *(Greenhalgh,2005) *[29]	Qualitative	A method developed from the need to synthesize evidence to inform complex policy-making questions, and involves looking across different paradigms/research traditions to uncover their ‘unfolding storyline” resulting in maps of ‘meta-narratives’ from which dimensions or themes can be revealed and distilled for the synthesis phase of the review.
**Meta-study** *(Paterson BL, 2001) *[30]	Qualitative	A multi-faceted, interpretive approach to synthesis developed to study the experiences of adults living with a chronic illness, and consists of 3 components to be done prior to synthesis: meta-data-analysis, meta-method, and meta-theory. Collectively, these create a new interpretation accounting for the results of all three elements of analysis.
**Meta-summary** *(Sandelowski M, 2003) *[31]	Qualitative	A quantitatively oriented summary of qualitative findings (as opposed to data being transformed) developed to accommodate the distinctive features of qualitative surveys. The approach includes the extraction, grouping, and formatting of findings, and the calculation of frequency and intensity effect sizes, which can be used to produce mixed research syntheses and to conduct ‘posteriori’ analyses of the relationship between reports and findings. Meta-summaries can serve as a basis for a further synthesis.
**Meta-synthesis** *(Sandelowski M, 1997) *[32]	Qualitative	A method developed in response to concerns about the relevance and utility of qualitative research, and involves combining separate elements to form a coherent whole using a process of logical deduction. Its aims are to portray an accurate interpretation of a phenomenon and to compare and contrast the constructs of individual studies to reach consensus on a new construction of that phenomenon. It involves: identifying findings, grouping findings into categories and grouping categories into synthesised findings.
**Mixed studies review** *(Pluye, 2005, Pluye 2009; Sandelowski M, 2003 book) *[33-35]	Mixed	A literature review that simultaneously examines qualitative, quantitative, and mixed methods primary studies to provide a greater understanding of a health issue than one type of research approach alone (including the process of searching, analysis and study quality appraisal).
**Narrative review / Narrative summary** *(Dixon-Woods M, 2005) *[36]	Mixed	An informal approach used to describe the selection, chronicling, and ordering of primary evidence to produce an account of the evidence with commentary and interpretation. It can ‘integrate’ qualitative and quantitative evidence through narrative juxtaposition (discussing diverse forms of evidence side by side). It is less concerned with assessing evidence quality and more focused on gathering relevant information that provides both context and substance to the authors’ overall argument.
**Narrative synthesis** *(Popay J, 2006) *[37]	Qualitative	Similar to “Narrative review”, it involves an approach to evidence review but includes a formal analytical process of synthesis to generate new insights or knowledge by seeking to be systematic and transparent. It involves the ‘simple’ juxtaposition of findings from the studies included in the review and some element of integration or interpretation. There are 3 main elements to the process: developing a preliminary synthesis of the findings of included studies; exploring relationships in the data; and assessing the robustness of the synthesis product.
**Qualitative cross-case analysis** *(Miles & Huberman. 1994; Yin R. 2003) *[38,39]	Mixed	Case studies are used to understand complex social phenomena. Research using a case study approach may be based on a single or multiple cases, and can include a mixture of qualitative and quantitative evidence.
**Qualitative meta- synthesis** *(Jensen & Allen 1996) *[40]	Qualitative	Meta-synthesis attempts to integrate results from a number of different but inter-related qualitative studies. The technique has an interpretive, rather than aggregating, intent, in contrast to meta-analysis of quantitative studies. Qualitative meta- synthesis defined as theories, grand narratives, generalizations, or interpretive translations produced from the integration or comparison of findings from qualitative studies.
**Qualitative systematic review / Qualitative evidence synthesis** *(Grant 2009) *[41]	Qualitative	Method for integrating or comparing findings from qualitative research. The method helps identify themes or constructs that lie in or across individual studies. The resulting accumulated knowledge may lead to the development of a new theory, an overarching “narrative” a wider generalization or “interpretative translation”.
**Quantitative case survey** *(Yin R and Heald K. 1975; Pelz D. 1981) *[42,43]	Mixed	A formal process for systematically coding data from a number of qualitative cases sufficient for quantitative analysis. A set of structured questions is used to extract data from individual case studies, which are then treated as observations within a single dataset. Data are then converted to quantitative form for statistical analysis. It is a way of turning qualitative studies into quantitative data for analysis, allowing an integrated qualitative-quantitative synthesis to be undertaken.
**Realist review / synthesis** *(Pawson 2005) *[10]	Mixed	Rooted in philosophy, this is a method used to investigate ‘what works for whom, under what circumstances, and why’. Primary focus is on the causal mechanisms or “theories” that underlie types of interventions or programmes and aims to build explanations across interventions or programmes which share similar underlying “theories of change” as to why they work (or not) for particular groups in particular contexts.
**Textual Narrative synthesis** *(Lucas, 2007) *[44]	Mixed	An approach that arranges studies into more homogeneous groups, and useful for synthesizing different types of evidence (quantitative, qualitative, economic, etc). Study characteristics, context, quality and findings are reported according to a standard format, and similarities and differences are compared across studies
**Thematic analysis** *(Mays, 2005) *[45]	Mixed	The most common method adopted within ‘Narrative reviews” to produce a relatively rudimentary synthesis of findings across the included studies. It involves identifying prominent or recurring themes in the literature (largely shaped by research questions), and summarizing the findings of different studies under thematic headings using summary tables, which can inform a description of key points.
**Thematic synthesis** *(Thomas, 2008) *[46]	Qualitative	This approach combines and adapts approaches from both meta-ethnography and grounded theory. Free codes of findings are organized into ‘descriptive’ themes, which are then further interpreted to yield ‘analytical’ themes (comparable to 3^rd^ order interpretations from meta-ethnography).

Some researchers have attempted to outline methods for the synthesis of qualitative [47] and mixed-methods research [36,45] and to build a typology of such reviews [41], while others have highlighted methods for knowledge synthesis reviews to inform specific end-user targets such as for management and policy-making in the health field [45]. A recent overview by Gough and colleagues attempted to outline the differences between review designs and methods by describing the important conceptual and practical differences amongst them [8]. However, a comprehensive manual for all of the different synthesis methods (quantitative/qualitative or mixed), outlining how they are related and how to decide which methodology is the most appropriate for a particular research question does not currently exist. To our knowledge, the current study will be the first to describe an overall taxonomy of all existing types of knowledge synthesis methods, to characterise the differences between them, and to develop a strategy for knowledge users to be able to select the most appropriate method to answer their research questions.

The specific objectives of the current study are to: (1) to conduct a systematic search for knowledge synthesis methods across multi-disciplinary fields, such as health and philosophy; (2) compare and contrast the different knowledge synthesis methods; and, (3) map out the specific steps to conducting the knowledge synthesis methods, which will be used to inform the development of a knowledge synthesis methods manual/tool.

## Methods/Design

### Search strategy

We will use the methodologically rigorous scoping review approach proposed by Arksey and O’Malley [48] to conduct a systematic search across the disciplines of health and philosophy. We will search the following electronic databases from inception onwards: MEDLINE, CINAHL, EMBASE, PsycInfo, the Cochrane Methodology Register, Cochrane Database of Systematic Reviews, Social Sciences Abstracts, LISA, Philosopher’s Index, and ERIC. We will also perform targeted searches for grey literature (i.e., difficult to locate or unpublished material) by searching 1) Google, 2) relevant discipline-based listservs (e.g., CANMEDLIB, MEDLIB), and 3) the websites of agencies that fund or conduct knowledge synthesis (e.g., CIHR, Canadian Agency for Drugs and Technologies in Health, Agency for Healthcare Research and Quality, Cochrane and Campbell Collaborations, Joanna Briggs Institute, Centre for Reviews and Dissemination).

The draft literature search for MEDLINE can be found in Additional file 1, which uses a combination of medical sub-headings (MeSH) and free text terms. It will be modified as necessary for the other databases. The search strategy will not be limited by study design, year or language of dissemination and will be peer reviewed by another information specialist using the Peer Review of Electronic Search Strategies (PRESS) checklist [49]. The literature search will be supplemented by scanning the reference lists of included studies, searching authors’ personal files, and contacting methodological experts in each field.

### Study selection: inclusion criteria

*Study design*: All study designs will be considered including qualitative and quantitative methods such as methodology reports; knowledge syntheses (including a description of the synthesis method); short reports describing the development, use, or comparison of methods for knowledge synthesis. *Type of knowledge synthesis*: We will focus on synthesis methods above and beyond traditional systematic reviews and exclude methods on economic analysis or clinical practice guidelines. *Disciplines*: Health: “*A state of complete physical, mental and social well-being and not merely the absence of disease or infirmity*” [50] (and thus includes the disciplines of psychology, education and sociology) and philosophy. These were selected because many of the knowledge synthesis methods originated from these disciplines (e.g., systematic review methods rooted in education and psychology; realist reviews based on philosophy).

### Study selection: screening

Prior to commencing the screening process, a calibration exercise will be conducted to ensure reliability in correctly selecting articles for inclusion. It will entail independently screening a random sample of 5% of the included citations by two reviewers. Eligibility criteria will be modified if low agreement is observed between the reviewers (e.g., a kappa statistic less than 50%). The reviewers will then independently screen the remainder of the search results using a pre-defined relevance criteria form for all levels of screening (e.g., title and abstract, full-text review). Discrepancies will be resolved by discussion with a third reviewer.

### Data abstraction

A data abstraction form will be tested independently by two reviewers on a random sample of 10 articles and revised iteratively, as needed. It is anticipated that the data items will include study characteristics (e.g., first author, year of publication) and characteristics related to the method (e.g., general description of the review method, discipline) (Additional file 1). Two investigators will independently read each article and extract the relevant data. Differences in abstraction will be resolved by discussion or the involvement of a third reviewer. We will not formally appraise methodological quality because the aim of a scoping review is to identify gaps in the evidence base and to target topic areas for future reviews.

### Data analysis

We will analyze the data according to a three-stage process aimed at addressing the three research objectives: to characterize the synthesis methodologies (Synthesis objective 1); to identify the similarities and differences amongst these methods (Synthesis objective 2); and to map out a process for conducting different synthesis methods and to provide an approach for matching the research question to the appropriate methods (Synthesis objective 3). Table 2 shows the analysis plan and anticipated outputs for each of these objectives. Data analysis will involve quantitative (e.g., frequency analysis) and qualitative (e.g., thematic analysis) methods. We anticipate that this multi-layer synthesis process will also identify existing gaps in the literature, and reveal potential topics for conducting other systematic or novel reviews in the future.

**Table 2:** Analysis plan and anticipated outputs for each of the 3 synthesis objectives

**Synthesis objective**	**Method**	**Questions to guide analysis**	**Anticipated outputs**
**1: To characterize the synthesis methodologies**	We will categorize or ‘chart’ [47] the synthesis methodology reported in each of the included studies using specific questions to guide the analysis	1. What is a general description of the knowledge synthesis method?	· To identify ‘x’ articles that report a knowledge synthesis method and of these, ‘y’ articles used the subjective idealism approach in ‘z’ discipline.
		2. What is the purpose of the knowledge synthesis method?	· A taxonomy of knowledge synthesis methods across multidisciplinary fields o Categorization of the synthesis methods to reveal what research is available within specific disciplines
		3.What is the epistemological approach of the method? Is it subjective idealism (i.e., there is no shared reality independent of multiple alternative human constructions) or objective idealism (i.e., there is a world of collectively shared understandings)?	
		4. Which discipline is the knowledge synthesis associated with (e.g., health, philosophy)?	
		5. What type of evidence can be synthesized by the knowledge synthesis method – quantitative, qualitative or mixed quantitative and qualitative?	
		6. How has the method been used to answer healthcare topics?	o Additional categories may be identified iteratively through completion of the search and in consultation with the team members including the knowledge users
**2: To identify the similarities and differences between these methods**	We will categorize articles that specifically address the similarities and differences between the knowledge synthesis methods by comparing the synthesis methodology reported in each of the included studies	1. What are the similarities and differences among the knowledge synthesis methods?	· An in-depth comparison of the review methods in a table including:
		2. How does the method differ from ‘traditional’ systematic review methods?	i. The specific features of the method that make it more appropriate to answer a question
		3. What is the minimum expertise required to implement the knowledge synthesis method? Are particular skills required? Is a particular disciplinary background recommended?	i. The facilitators and barriers to using one synthesis method over another *(especially if more than one synthesis method may be appropriate to answer the same research question)*
		4. What are the advantages and disadvantages of each knowledge synthesis method?	
		5. How comprehensive is the knowledge synthesis method? Can it be used for the entire synthesis or only for a part of the synthesis (e.g., the analysis)?	
		6. How applicable is the method and how can it be applied to healthcare interventions?	
**3: To map out a process for conducting different synthesis methods and to provide an approach for matching the appropriate method to answer research questions**	We will categorize key articles that explicitly explain the specific methodology of the knowledge synthesis method.	1. What are the specific steps to conducting the knowledge synthesis method?	· An algorithm to guide synthesis methodology *(informed by findings from objective 2 and consultation with knowledge users)*
		2. Was the method empirically derived (i.e., through experiment and observation) or theoretically derived?	· The mapping of specific steps to conducting the review · A bibliography of articles that describe how to conduct the different knowledge synthesis methods
		3. Are the steps operationalized (i.e., reported in a reproducible manner)?	
		4. In what disciplinary fields and contexts are the steps operationalizable? Can they feasibly be applied to other contexts?	

### Engagement of knowledge users and KT plan

We have adopted an integrated KT approach to this project through the inclusion of knowledge users (i.e., systematic review methodologists, journal editors, review funders, policy makers, students and educators who teach knowledge synthesis methodology), who have been and will continue to be involved in every step of the process through to the reporting format and the methods for disseminating and implementing findings, drawing on Graham’s Knowledge-to-Action (KTA) framework [51]. We plan to develop an active KT plan by: 1) identifying the key messages arising from this research project; 2) determining the principal target audiences for each of these messages; 3) seeking out the most credible messenger for these messages and engaging their interest in becoming involved in the communication of these messages; and 4) launching a KT strategy grounded in the best available research evidence. We will use a diverse range of approaches to disseminate the results of this review to the different stakeholder groups (including an interactive workshop that will bring together the key target audiences for our research). These strategies will ensure that the research continues to reflect the relevant needs of the end users of this information, and to facilitate appropriate dissemination of outputs.

### Anticipated challenges

We foresee some potential challenges related to this scoping review. First, the yield of the literature search might be more extensive than anticipated—the team will work closely with the information specialist to ensure that the scope is manageable. Second, it might be challenging to categorize the knowledge synthesis methods accurately (e.g., distinguishing between quantitative/qualitative or mixed / hybrid approaches or those not formally categorized) and to appropriately match a research question with a synthesis method. However, we have a strong team with diverse experience in different research methods, and are planning to hold stakeholder meetings to iteratively receive in-depth feedback from our end users.

## Discussion

The proposed scoping review has the potential to impact practice and policy and will make several contributions to the KT and health services research literature. First, the work will advance the science of knowledge synthesis by providing a systematic process for key knowledge users to make informed decisions about which synthesis method is the most appropriate to answer their research questions. This may also augment the quality of the research evidence produced. In particular, the work will highlight the potential for novel knowledge synthesis methods to clarify complex, multi-component, and multi-disciplinary healthcare interventions [13], and to contribute to the advancement of evidence-based practice and evidence-based decision-making. Second, there is currently no comprehensive manual for all available synthesis methods (quantitative/qualitative or mixed). To develop this manual, a taxonomy and comparison of all available synthesis methods are needed. Our work aims to develop the taxonomy of synthesis methods across multiple disciplines such as health and philosophy. Third, the scoping review will help map the literature, identify gaps where primary methods evidence is lacking and needed, and where systematic reviews are required; we anticipate that this work will lead to multiple subsequent systematic reviews. For example, one future systematic review may focus on knowledge synthesis methods for health services research and another may focus on knowledge synthesis of qualitative data. Fourth, the work has the potential to directly influence knowledge synthesis funders such as the Canadian Institutes of Health Research (CIHR) in developing resources (e.g., modules) that can be used to increase awareness of novel synthesis methods and their relevance for addressing complex evidence. This information is especially imperative for those conducting peer review of knowledge synthesis grants. Fifth, the scoping review can be used by publishers and editors to assist with the peer review of manuscripts describing these types of knowledge syntheses. Sixth, our findings have the potential to influence health research methods curricula within clinical epidemiology programs, by expanding the current understanding of synthesis methods. The development and evaluation of complex interventions has emerged as an important component of KT, so expertise in conducting non-traditional review methods will become increasingly important for researchers, teachers, and students. Lastly, the work will be targeted across a broad scope of health disciplines, which will provide the opportunity to elicit more generalizable findings that can directly inform practice and policy decisions within these disciplines. Results from this work will be the starting point of a comprehensive manual and decision algorithm on how to conduct the different synthesis methods and the proposed KT strategy will serve to engage the relevant stakeholders in clarifying and fulfilling the research agenda proposed in the scoping review (Table 3 summarizes the anticipated products that will be generated).

**Table 3:** Anticipated products generated by the scoping review

**Product**	**Audience**	**Method**
**Taxonomy for review methods**	Health services researchers, trainees	Publish in relevant journals; present at relevant academic meetings (e.g. Cochrane Colloquium); provide the taxonomy online through the Knowledge Synthesis Network, KT Canada, Cochrane Collaboration, CIHR.
**Algorithm for matching the synthesis question to the appropriate review method(s)**	Health services researchers, trainees, publishers, journal editors, funders	Prepare summary document describing the algorithm that will be disseminated through publication in relevant journal(s). Provide the algorithm online through the Knowledge Synthesis Network, KT Canada, Cochrane Collaboration, CIHR.
**Methods Manual**	Health services researchers, funders, publishers, and policy makers, and trainees	Develop online methods manual outlining the different review methods to be available as a series of articles, a set of powerpoint slides, and podcasts. We will also explore making these available as a book and have had preliminary discussions with Wiley Blackwell about this topic. Create an online systematic review course.
**Review Course**	Researchers and trainees	Create an online systematic review course to provide instruction in the methods for completing less traditional knowledge synthesis.

Abbreviations: *KT* knowledge translation; *CIHR* Canadian Institutes of Health Research.

Conducting a scoping review of available knowledge synthesis methods across multi-disciplinary fields will help funders, publishers, policy-makers, researchers, teachers, and students make informed decisions about the most appropriate synthesis method to answer research questions about complex evidence, and provide the opportunity to elicit findings directly informing practice and policy decisions.

## Competing interests

The authors declare that they have no competing interests.

## Authors’ contributions

All authors participated in the design and development of the protocol. MK, ACT, and SES drafted the manuscript, and all authors read and approved the final manuscript.

## Funding

The study was funded by a Canadian Institutes of Health Research (CIHR) Knowledge Synthesis grant. MK holds a CIHR Banting Postdoctoral Fellowship, ACT a CIHR/DSEN new investigator award, and SES a Tier 1 Canada Research Chair in Knowledge Translation.

## Pre-publication history

The pre-publication history for this paper can be accessed here:

http://www.biomedcentral.com/1471-2288/12/114/prepub

## Supplementary Material

Additional file 1 Appendices. Click here for file
