# Supplementary material for: What is the most appropriate knowledge synthesis method to conduct a review? Protocol for a scoping review
Source: BMC Med Res Methodol. 2012 Aug 3;12:114. doi: 10.1186/1471-2288-12-114 (PMC3477082; doi:10.1186/1471-2288-12-114)
Supplement: Additional file 1 — Appendices. [file 1471-2288-12-114-S1.doc]

**APPENDIX A:**

MEDLINE Literature Search Strategy

Database: Ovid MEDLINE(R), Ovid MEDLINE(R) In-Process & Other Non-Indexed Citations, Ovid MEDLINE(R) Daily and Ovid OLDMEDLINE(R) <1946 to Present>

Search Strategy:

--------------------------------------------------------------------------------

1. (cochrane adj2 (method? or methodolog$ or process$ or procedure? or development$ or technique?)).tw.
2. ("comprehensive review?" adj2 (method? or methodolog$ or process$ or procedure? or development$ or technique?)).tw.
3. ("critical review?" adj2 (method? or methodolog$ or process$ or procedure? or development$ or technique?)).tw.
4. ("evidence review?" adj2 (method? or methodolog$ or process$ or procedure? or development$ or technique?)).tw.
5. ("evidence overview?" adj2 (method? or methodolog$ or process$ or procedure? or development$ or technique?)).tw.
6. ("evidence synthes?s" adj2 (method? or methodolog$ or process$ or procedure? or development$ or technique?)).tw.
7. ("integrative review?" adj2 (method? or methodolog$ or process$ or procedure? or development$ or technique?)).tw.
8. ("integrative overview?" adj2 (method? or methodolog$ or process$ or procedure? or development$ or technique?)).tw.
9. ("integrative synthes?s" adj2 (method? or methodolog$ or process$ or procedure? or development$ or technique?)).tw.
10. ("literature review?" adj2 (method? or methodolog$ or process$ or procedure? or development$ or technique?)).tw.
11. ("literature overview?" adj2 (method? or methodolog$ or process$ or procedure? or development$ or technique?)).tw.
12. ("literature synthes?s" adj2 (method? or methodolog$ or process$ or procedure? or development$ or technique?)).tw.
13. (meta-analys?s adj2 (method? or methodolog$ or process$ or procedure? or development$ or technique?)).tw.
14. (metaanalys?s adj2 (method? or methodolog$ or process$ or procedure? or development$ or technique?)).tw.
15. (metanalys?s adj2 (method? or methodolog$ or process$ or procedure? or development$ or technique?)).tw.
16. (meta-synthes?s adj2 (method? or methodolog$ or process$ or procedure? or development$ or technique?)).tw.
17. (metasynthes?s adj2 (method? or methodolog$ or process$ or procedure? or development$ or technique?)).tw.
18. ("methodologic$ review?" adj2 (method? or methodolog$ or process$ or procedure? or development$ or technique?)).tw.
19. ("methodologic$ overview?" adj2 (method? or methodolog$ or process$ or procedure? or development$ or technique?)).tw.
20. ("methodologic$ synthes?s" adj2 (method? or methodolog$ or process$ or procedure? or development$ or technique?)).tw.
21. ("qualitative review?" adj2 (method? or methodolog$ or process$ or procedure? or development$ or technique?)).tw.
22. ("qualitative systematic review?" adj2 (method? or methodolog$ or process$ or procedure? or development$ or technique?)).tw.
23. ("qualitative overview?" adj2 (method? or methodolog$ or process$ or procedure? or development$ or technique?)).tw.
24. ("qualitative synthes?s" adj2 (method? or methodolog$ or process$ or procedure? or development$ or technique?)).tw.
25. ("qualitative evidence synthes?s" adj2 (method? or methodolog$ or process$ or procedure? or development$ or technique?)).tw.
26. ("quantitative review?" adj2 (method? or methodolog$ or process$ or procedure? or development$ or technique?)).tw.
27. ("quantitative overview?" adj2 (method? or methodolog$ or process$ or procedure? or development$ or technique?)).tw.
28. ("quantitative synthes?s" adj2 (method? or methodolog$ or process$ or procedure? or development$ or technique?)).tw.
29. ("rapid review?" adj2 (method? or methodolog$ or process$ or procedure? or development$ or technique?)).tw.
30. ("scoping review?" adj2 (method? or methodolog$ or process$ or procedure? or development$ or technique?)).tw.
31. ("scoping stud$" adj2 (method? or methodolog$ or process$ or procedure? or development$ or technique?)).tw.
32. ("systematic review?" adj2 (method? or methodolog$ or process$ or procedure? or development$ or technique?)).tw.
33. ("systematic overview?" adj2 (method? or methodolog$ or process$ or procedure? or development$ or technique?)).tw.
34. ("systematic search and review" adj2 (method? or methodolog$ or process$ or procedure? or development$ or technique?)).tw.
35. ("campbell collaboration" adj2 (method? or methodolog$ or process$ or procedure? or development$ or technique?)).tw.
36. *"Review Literature as Topic"/ and (method? or methodolog$ or process$ or procedure? or development$ or technique?).tw.
37. *Meta-Analysis as Topic/ and (method? or methodolog$ or process$ or procedure? or development$ or technique?).tw.
38. "critical interpretive synthes?s".tw. [ evolving methods ]
39. (mapping adj review?).tw.
40. meta-ethnograph$.tw.
41. metaethnograph$.tw.
42. meta-interpretation?.tw.
43. metainterpretation?.tw.
44. meta-narrative?.tw.
45. metanarrative?.tw.
46. meta-stud$.tw.
47. metastud$.tw.
48. meta-summar$.tw.
49. metasummar$.tw.
50. "mixed studies review?".tw.
51. "mixed methods review?".tw.
52. (realist adj review?).tw.
53. (realist adj synthes?s).tw.
54. "state-of-the-art review?".tw.
55. "state of the art review?".tw.
56. "systematic literature survey?".tw.
57. "systematized review?".tw.
58. teleoanalys?s.tw.
59. or/1-58
60. "cochrane database of systematic reviews".jn.
61. 59 not 60

**APPENDIX B:**

Draft Data Abstraction Form

**Study Characteristics**

1. First author and year of publication
2. Reference ID number
3. Study design
4. Publication type
5. Focus of the paper
6. Year of publication
7. Country
8. Knowledge synthesis method
9. Discipline

**Objective 1:**

1. General description of knowledge synthesis method
2. Purpose of the knowledge synthesis method
3. Epistemological approach of the knowledge synthesis method (subjective idealism - there is no shared reality independent of multiple alternative human constructions versus objective idealism - there is a world of collectively shared understandings)
4. Type of evidence that can be synthesized by the knowledge synthesis method (quantitative, qualitative or mixed quantitative and qualitative)
5. How the method was used to answer a research question within healthcare (if relevant)

**Objective 2:**

1. Describe any similarities and differences between this knowledge synthesis method and other knowledge synthesis methods
2. Describe how the method differs from the ‘traditional’ systematic review methodology
3. Describe the minimum expertise required to implement the knowledge synthesis method including the skills required and recommended disciplinary background
4. List the advantages and disadvantages of each knowledge synthesis method
5. Describe the comprehensiveness of the knowledge synthesis method (used for entire synthesis versus only for a part of the synthesis method)
6. Applicability of the method to healthcare interventions

**Objective 3**:

1. Is this a key article that explicitly explains a complementary and alternative knowledge synthesis method? YES NO
2. If yes, please write the full citation of the article
3. What are the specific steps to conducting the knowledge synthesis method?
4. Was the method empirically derived (i.e., through experiment and observation) or theoretically derived?
5. Are the steps operationalized (i.e., reported in a reproducible manner)?
6. In what disciplinary fields and contexts are the steps operationalizable? Can they feasibly be applied to other contexts?
